# Medical Society of the County of Erie

**Published:** 1906-05

**Authors:** Franklin C. Gram


### Medical Society of the County of Erie.

### Special Meeting, April 3, 1906.

Reported By FRANKLIN C. GRAM, M. D., Secretary.

A special meeting of the Medical Society of the County of Erie was held in the rooms of the Buffalo Society of Natural Sciences, at 4:30 P. M., April 3, 1906, the president, Dr. A. H. Briggs, in the chair.

The purpose of the meeting was to consider Assembly bill No. 1715. Dr. A. G. Bennett, chairman of the committee on legislation, read the bill to the Society and then offered the following preamble and resolutions:

Whereas, Assembly bill No. 1715 has been read in our hearing at this meeting and,

Whereas, We find the provisions of said bill No. 1715 to be, according to our best judgment, in harmony with the policy of the State during its entire history, in the interests of the people and of the Public Health,

Therefore, Resolved, That the Medical Society of the County of Erie hereby endorses Assembly bill No. 1715 and congratulates the Assembly Committee on Public Health, upon its wisdom in constructing and introducing this important measure.

Resolved, That the Medical Society of the County of Erie commends Assembly bill No. 1715 to the favorable consideration of the Senators and Members of Assembly of the County and requests them to use all honorable means to promote its passage.

Resolved, That copies of these resolutions be sent to the Senators and Members of Assembly representing the County of Erie, to the committee on public health, and that copies be offered to the daily press.

In seconding the resolution Dr. H. R. Hopkins made a brief analysis of the most important points. The bill, he said. reduces the number of state examiners from 21 to 9; takes from the State societies the privilege of nominating members to the board of examiners; it is not the work of any medical society, county or state, but of laymen, or rather the Committee on public health of the Assembly. It defines the practise of medicine in a clear and precise manner, thereby setting aside all other definitions, especially the Daniel's decision, which many consider an absurd interpretation of the dark ages; it gives greatly needed power to regulate the practise of quacks, and finally, effectually disposes of the numerous schools in medicine which have spread like a contagion.

Dr. Charles G. Stockton expressed satisfaction at the appearance of the bill and approved of the resolutions offered, but while endorsing it as a whole he thought certain parts might be improved. For example, there are to be nine examiners and nine subjects, the evident intent being not to introduce any subject which might start a controversy between schools. It seemed like folly to leave out medicine from the examination of men who are to practise medicine. He thought the fees from examinations should go to the examiners, while the secretary should be paid by the State. He offered the following amendment to Dr. Bennett's resolution:

"This Society respectfully advises that the salary of the secretary of the Board of Examiners should be paid by the State, and that the fees received for examinations should be set apart for the compensation of the Examiners. It is further the belief of this Society, and it respectfully recommends to the Legislature, that the list of subjects upon which examinations should be held would be better arranged as follows: Anatomy, Physiology, Chemistry, Pathology, Principles of Medicine and Diagnosis, Surgery, Obstetrics and Gynecology, Hygiene and Bacteriology, and Materia Medica."

Dr. A. A. Hubbell said the bill appealed to him most favorably. He had carefully considered it. One of its purposes is to unify the examination process. It leaves out the question of schools as to therapeutics, a topic not essential to qualification in the practise of medicine. The colleges look after that part of the medical education, and of necessity keep up with medical progress and times. It raises the standard throughout the State, and specifies the length of preparation and how. There will always be practitioners whose character is such that they ought not to be permitted to practise, but at present there is no way to annul their license or authority, which is contemplated by the new bill.

Dr. Howe agreed with the suggested changes but considered it advisable to approve the bill in its present form, rather than jeapordize its passage by proposed amendments.

Similar opinions were expressed by Drs. Grosvenor, Krauss, and Clark.

Dr. Stockton's amendment, not being seconded, the original motion was unanimously adopted, whereupon the meeting adjourned.

